# Supplementary material for: Changes in Gut Bacterial Translation Occur before Symptom Onset and Dysbiosis in Dextran Sodium Sulfate-Induced Murine Colitis
Source: mSystems. 2021 Dec 7;6(6):e00507-21. doi: 10.1128/mSystems.00507-21 (PMC8651081; doi:10.1128/mSystems.00507-21)
Supplement: FIG S1 [file msystems.00507-21-sf001.pdf]

A

Day	-3	-2	-1	0	1	2	3	4	5	6	7	8	9	10	17	21
Body weight	●	●	●	✓	✓	✓	✓	✓	✓	✓	✓	✓	✓	✓		
LCN-2	●	●	●	●	●	●	●	●	●	●	●	●	●	●		
Blood in stool					✓	✓	✓	✓	✓	✓	✓	✓	✓	✓		
% HNA, LNA, PI	○	●	●	●	●	✓	✓	✓	✓	✓	✓	✓	✓	✓	●	●
% BONCAT	○	●	○	●	○	○	✓	✓	✓	✓	✓	○	○	✓	✓	✓
16S whole community	○	●	●	●	○	○	✓	✓	✓	✓	✓			✓		
16S BONCAT	○	●	○	●	○	○	✓	✓	✓	✓	✓	○	○	✓	●	●
16S sorted fractions	○	●	●	●	○	○	●	●	✓	✓	✓	○	○	✓	●	●

B

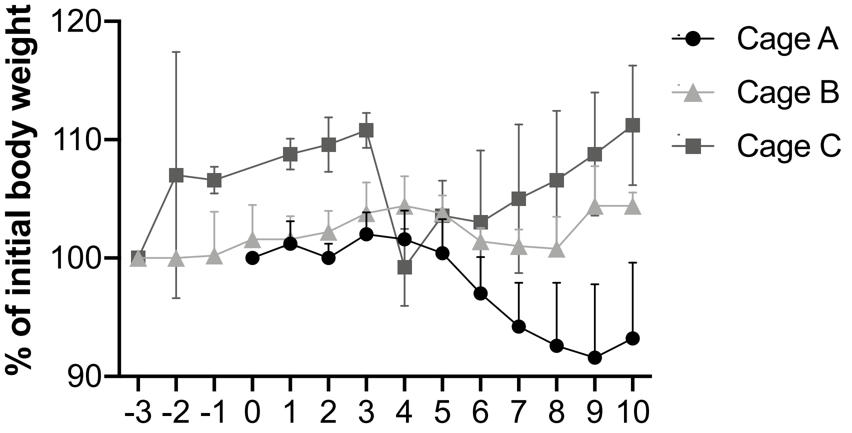

C

Cage	Mouse	Day											
		1	2	3	4	5	6	7	8	9	10	11	12
A	M1	+	+				+	+	+	+			
	M3			+		+	+	+		+			
	M5				+		+	+	~				
	M7						+	+	+		+	+	
	M9												
B	M2			+	+	+	+	~					
	M4			+	+	+	+	~					
	M6				+	+	+	+					
	M8				+	+	+	+					
	M10				+	+	+	~					
C	M11				+	+	+	+	~				
	M13					+	+	+					
	M15				+	+	+						
	M17				+	+	+						
	M19			+	+	+	~						

+ = positive for blood in stool
~ = inconclusive
