# Supplementary figures and images for: Changes in Gut Bacterial Translation Occur before Symptom Onset and Dysbiosis in Dextran Sodium Sulfate-Induced Murine Colitis
Source: mSystems. 2021 Dec 7;6(6):e00507-21. doi: 10.1128/mSystems.00507-21 (PMC8651081; doi:10.1128/mSystems.00507-21)

**A**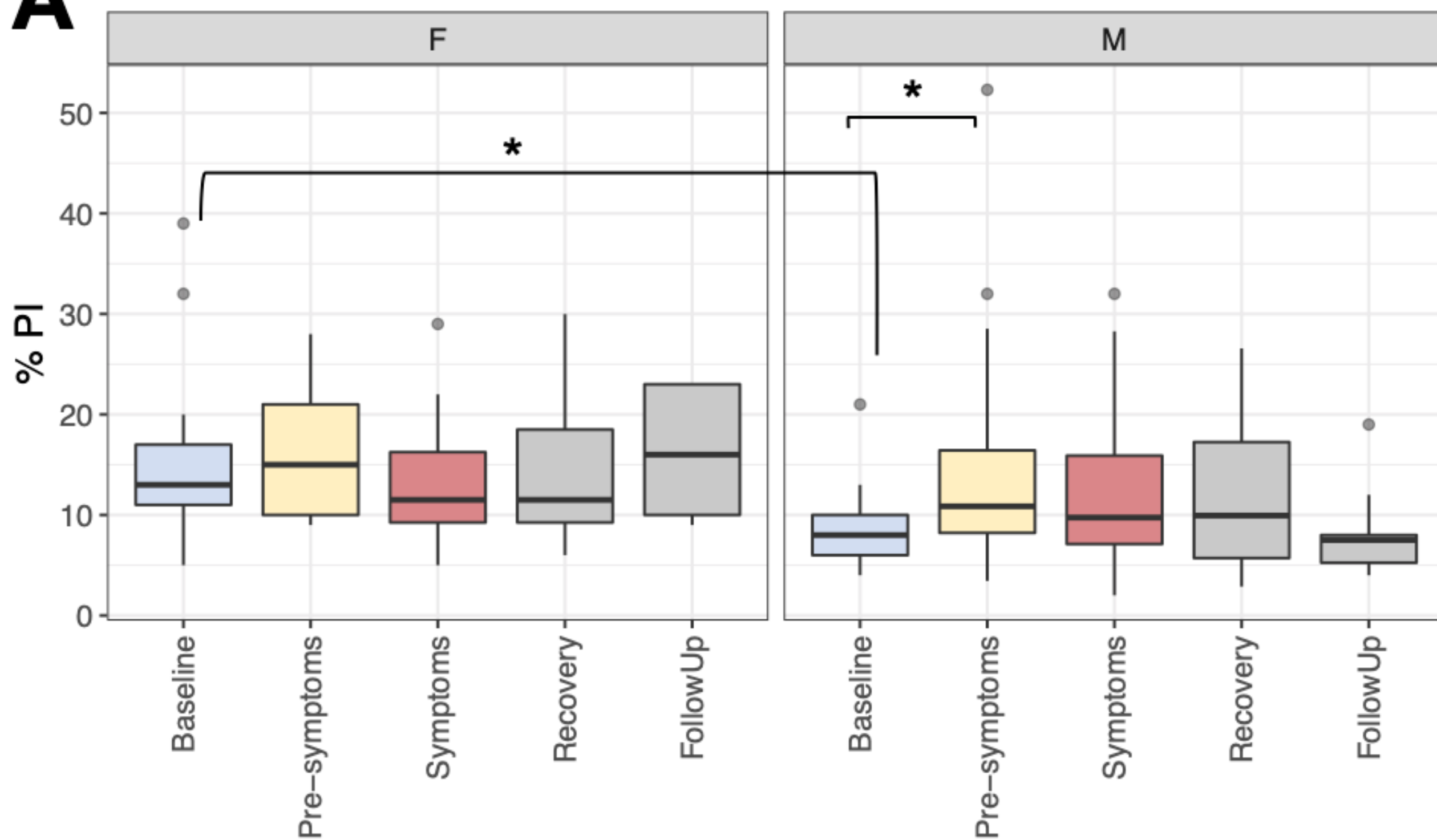**B**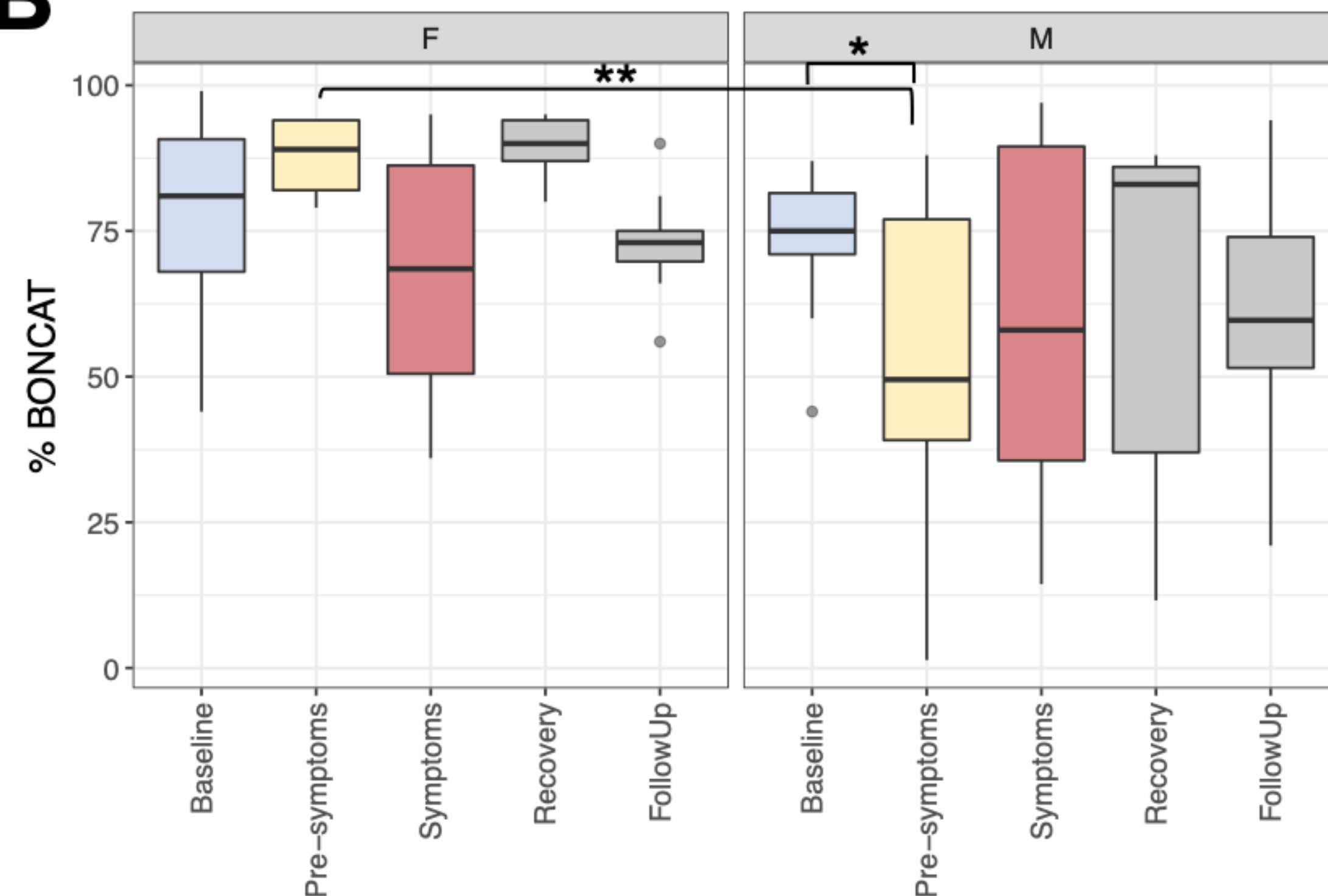**C**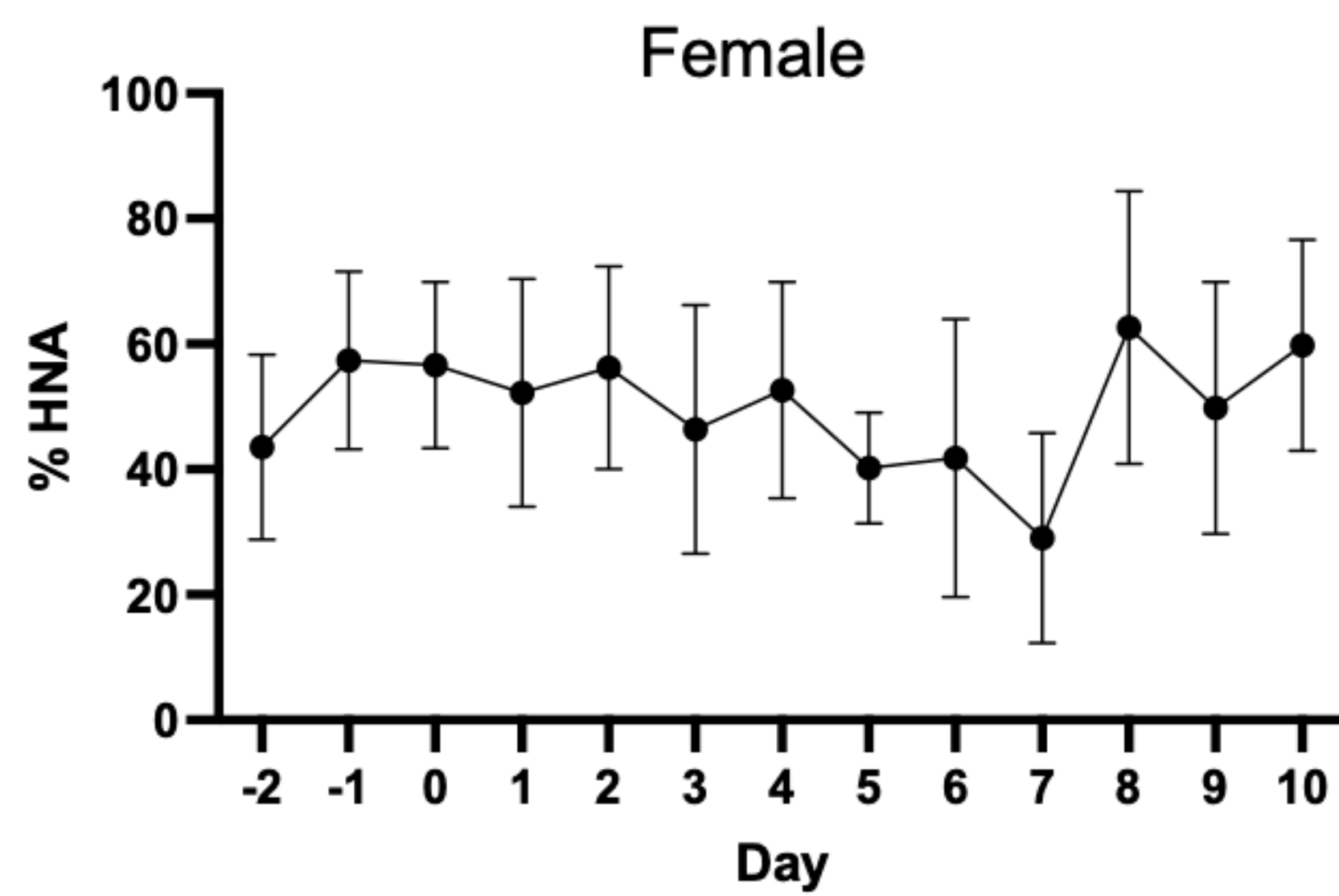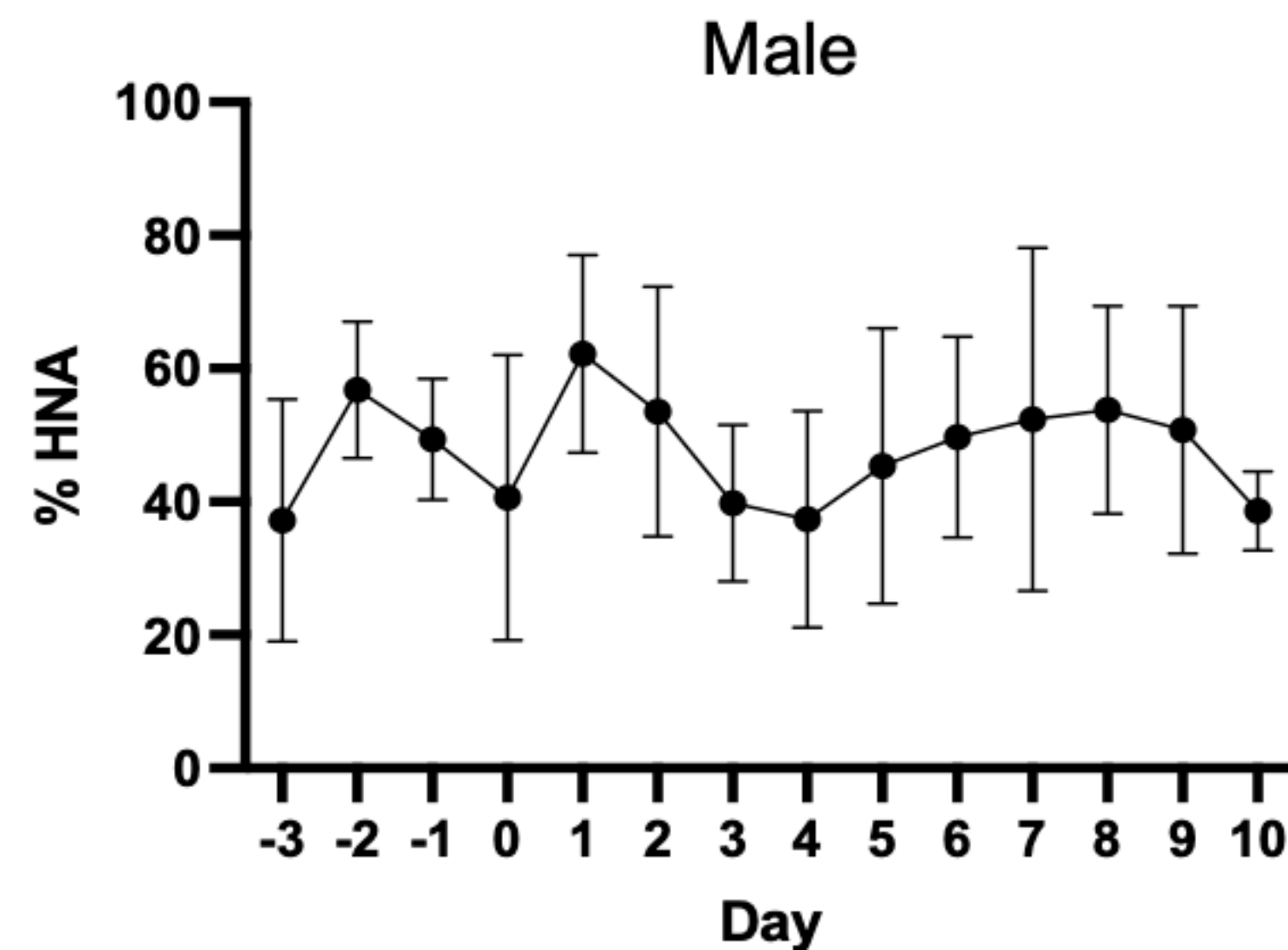

Supplement: FIG S2 [file msystems.00507-21-sf002.pdf]

A

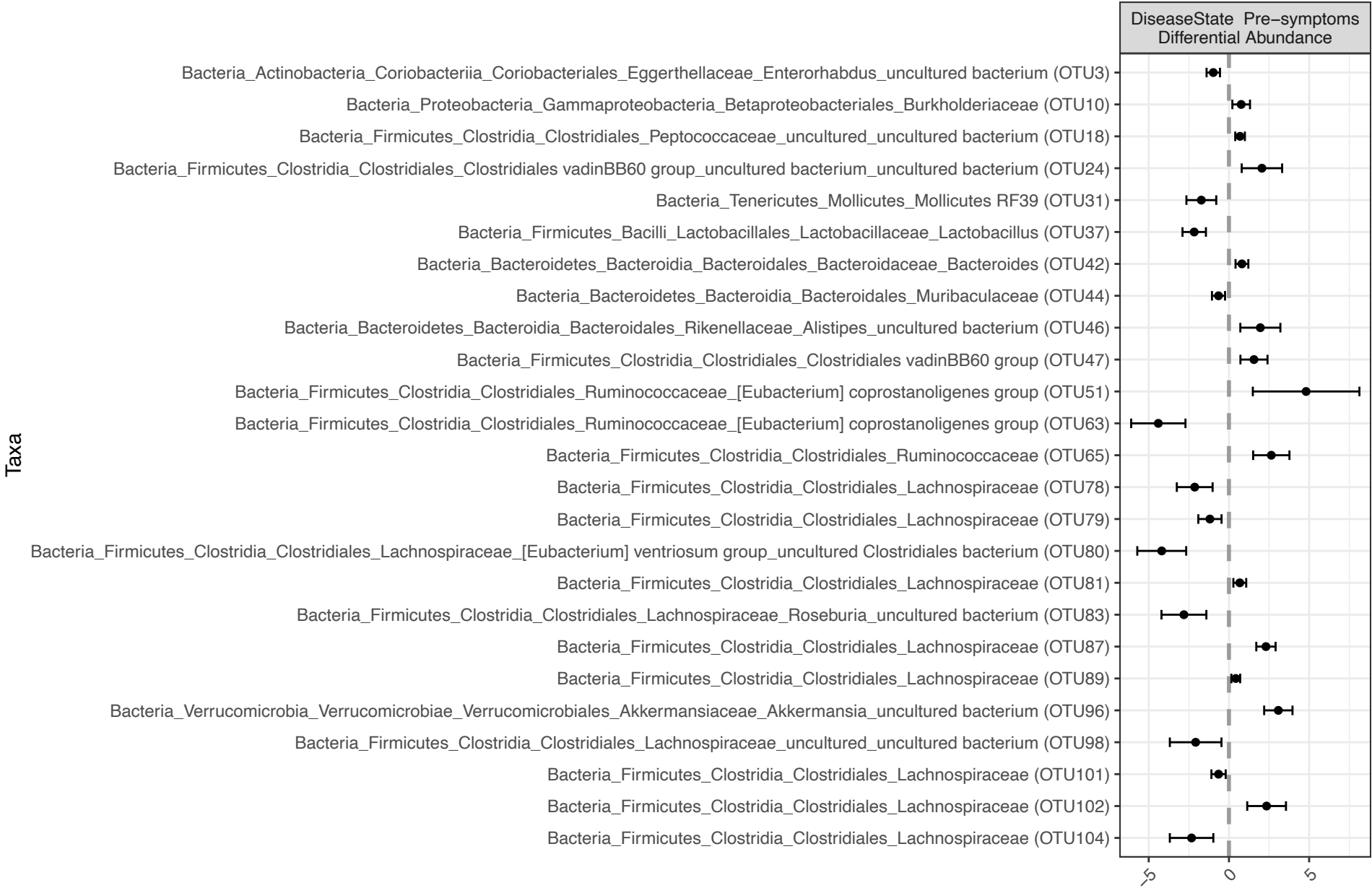

B

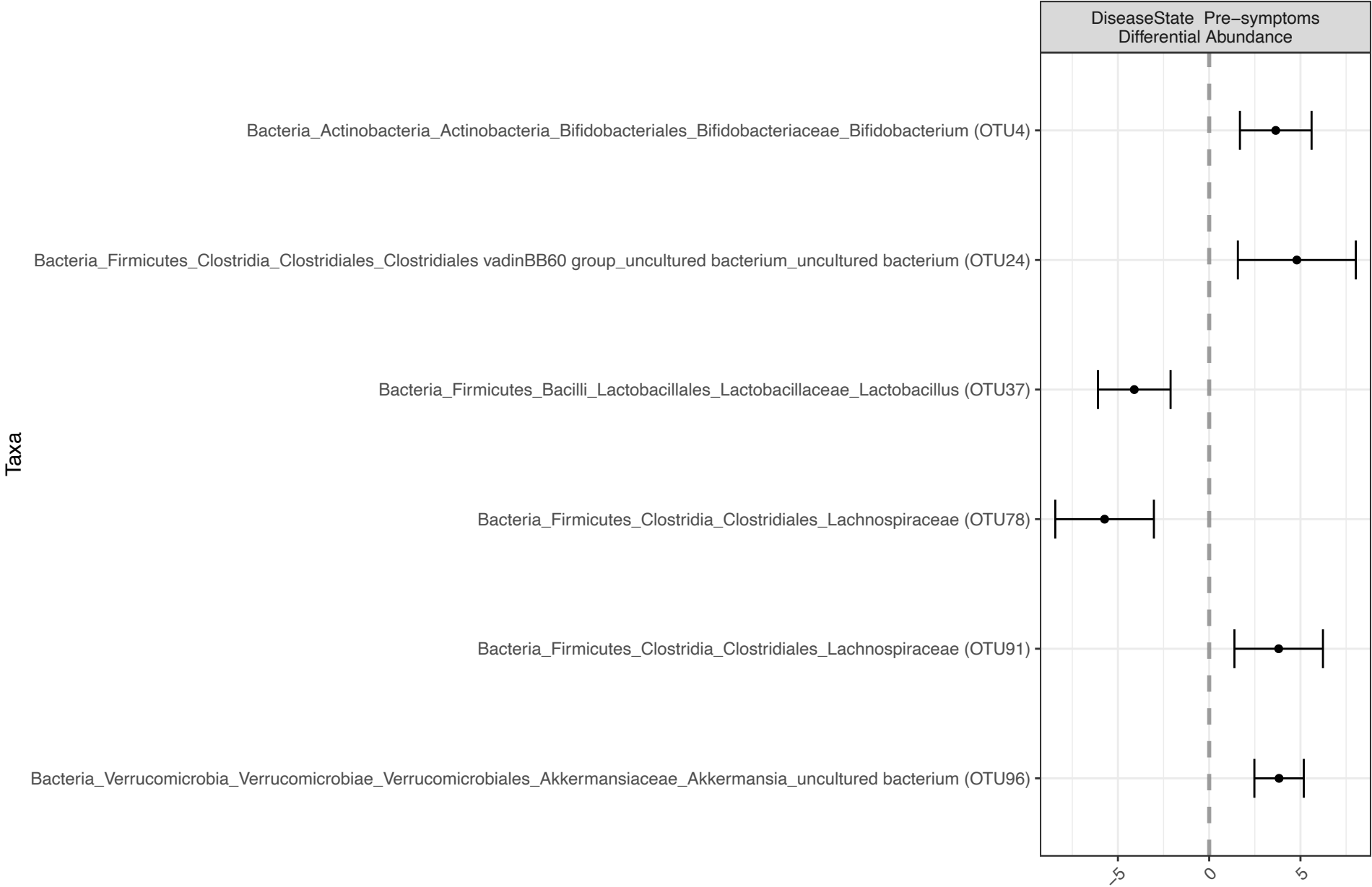

C

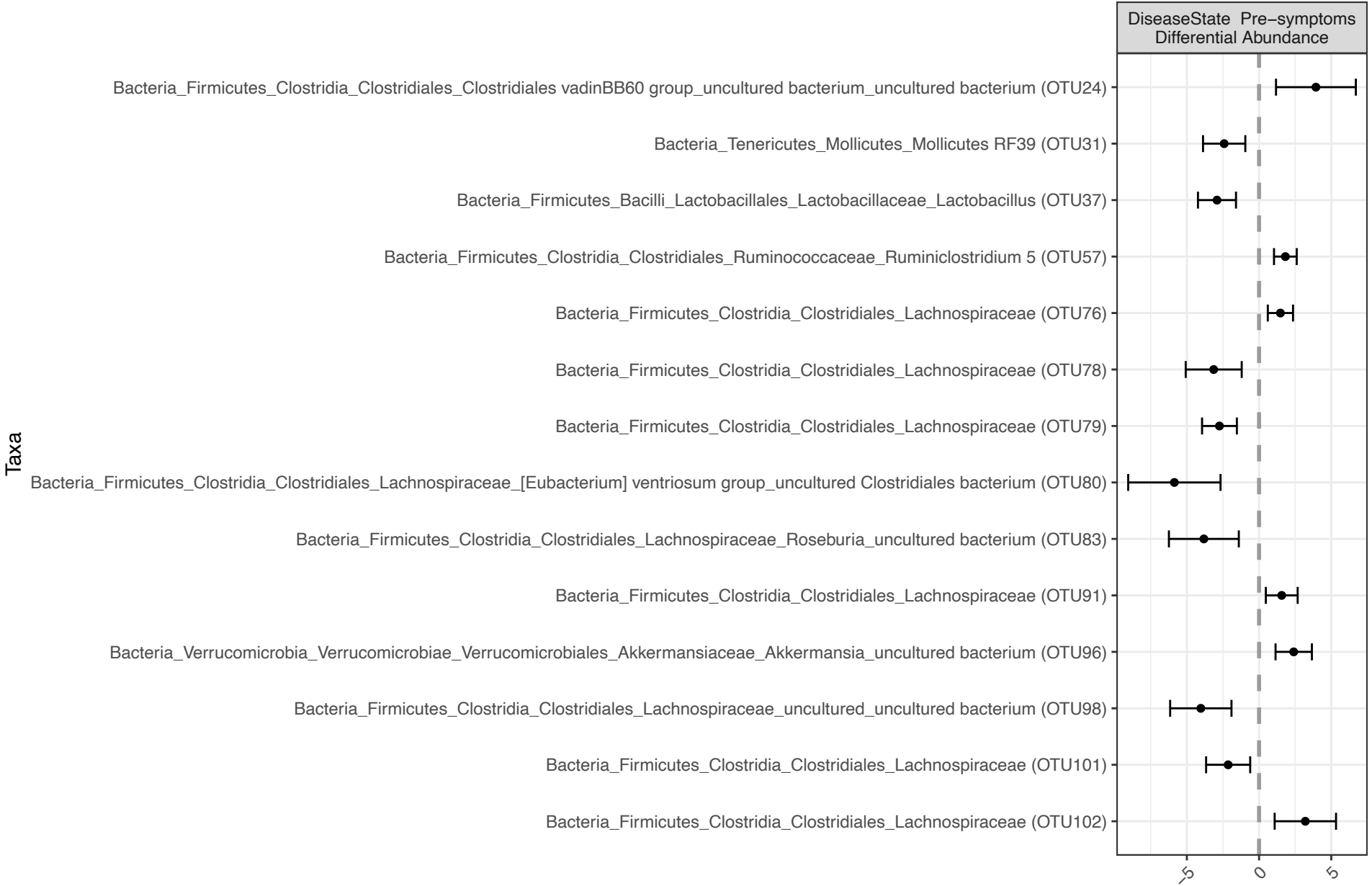

D

Taxa

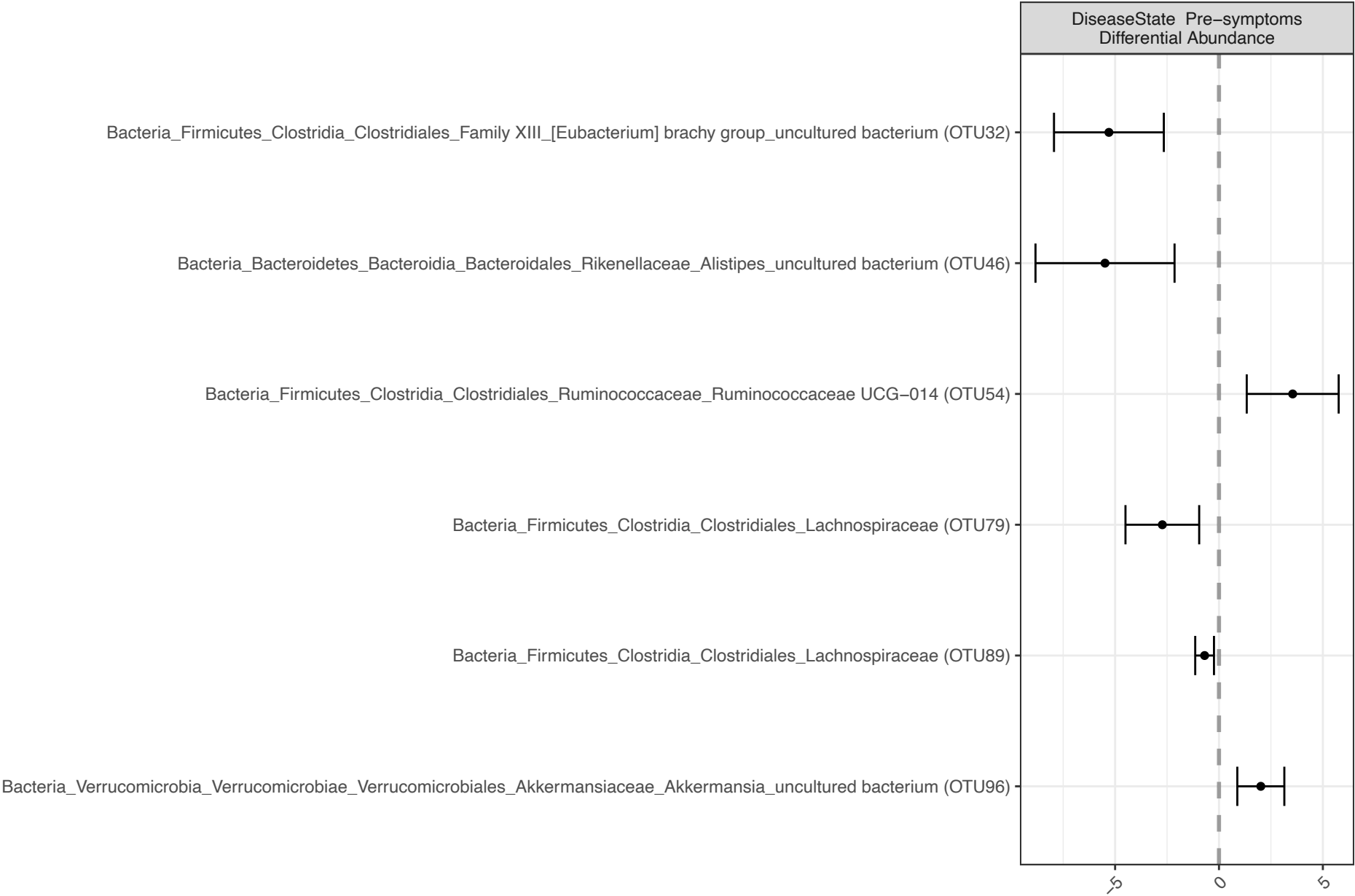

E

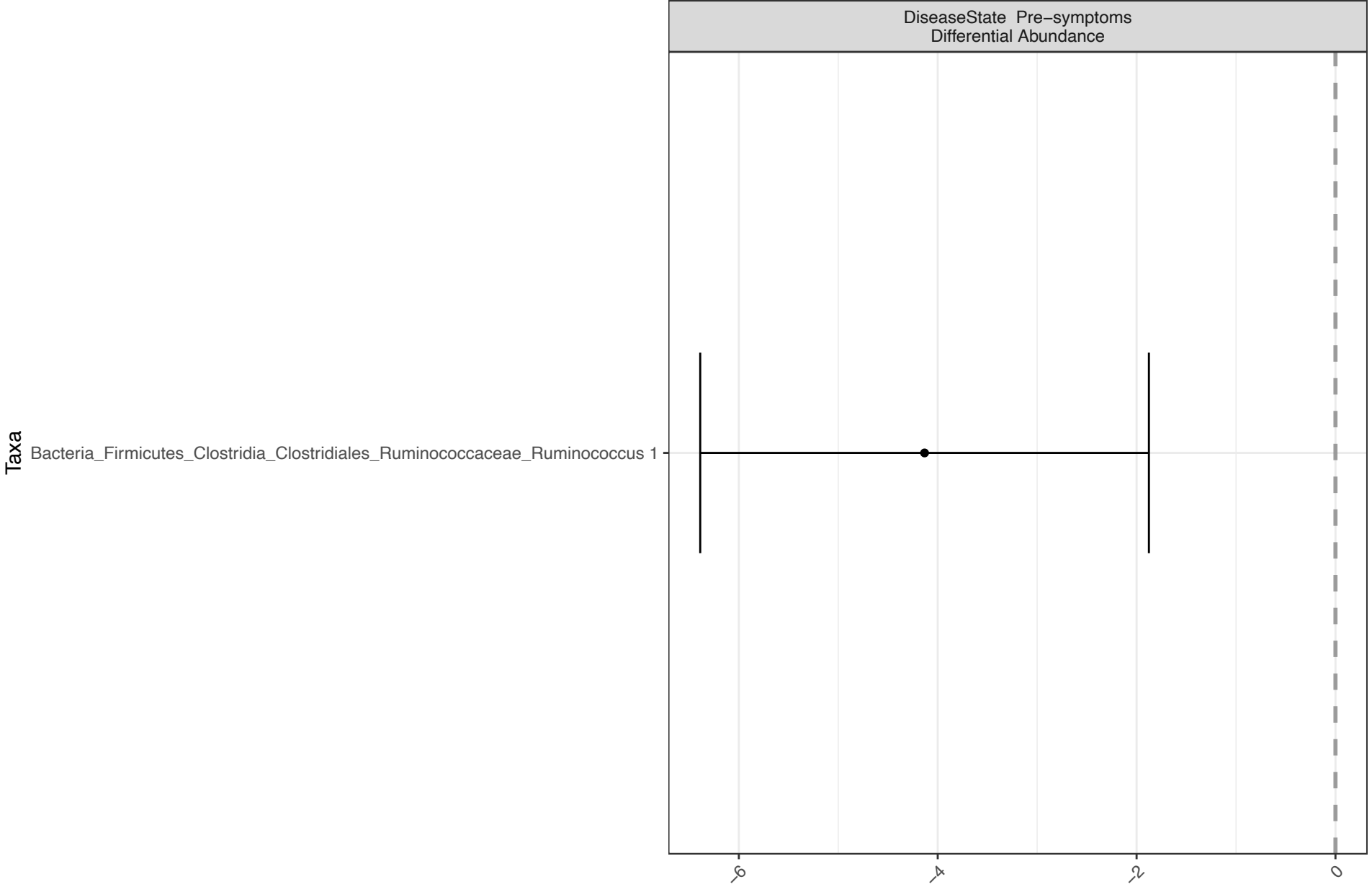

Supplement: FIG S3 [file msystems.00507-21-sf003.pdf]

**A**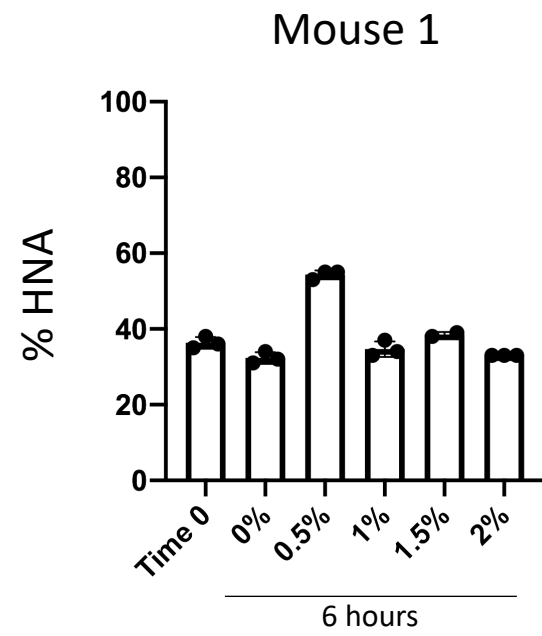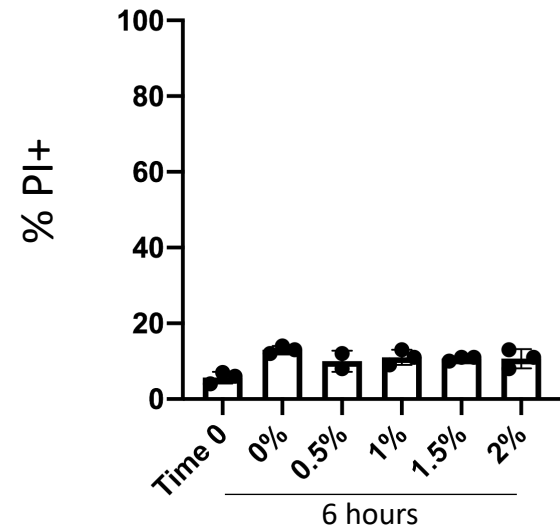

Mouse 2

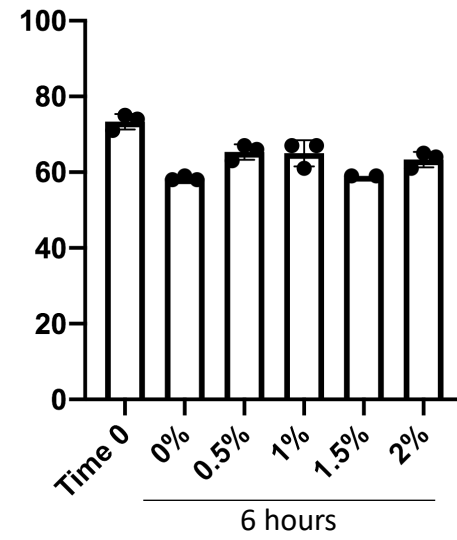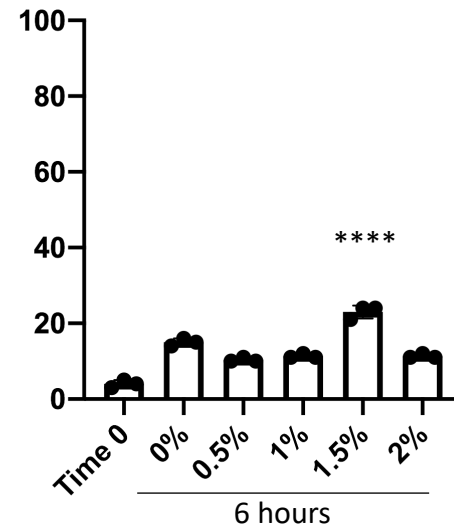**B**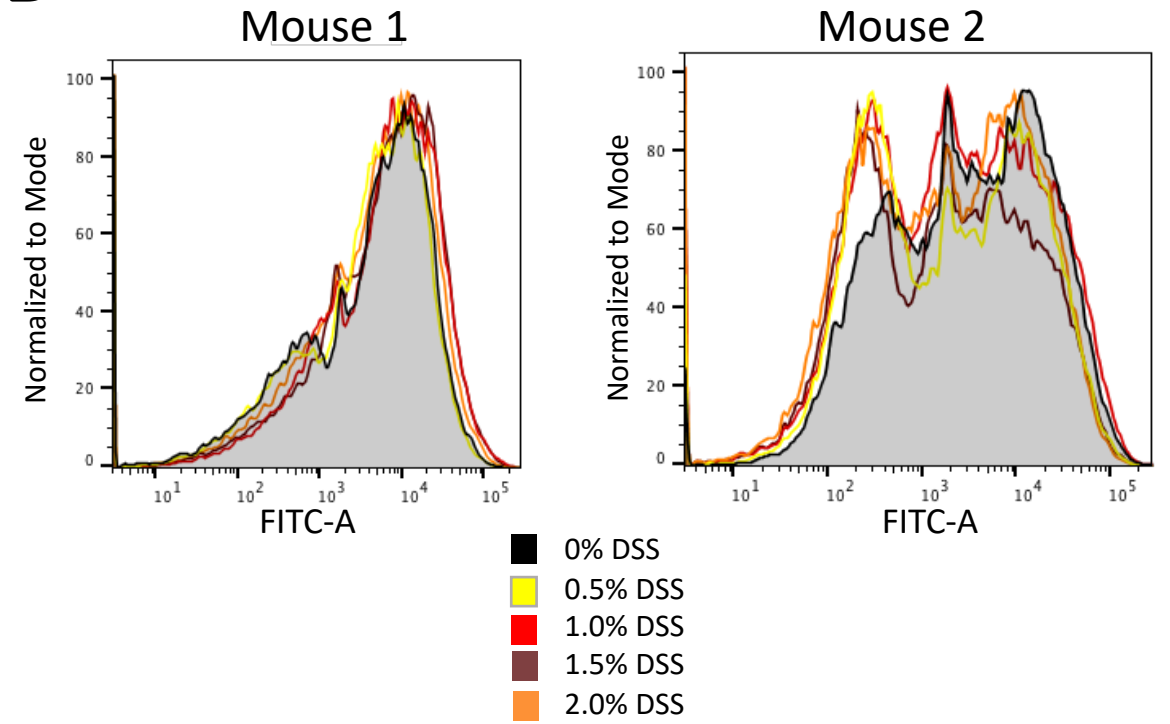

Supplement: FIG S4 [file msystems.00507-21-sf004.pdf]
